# Red cell distribution width at hospital discharge and out-of hospital outcomes in critically ill non-cardiac vascular surgery patients

**DOI:** 10.1371/journal.pone.0199654

**Published:** 2018-09-05

**Authors:** Gerdine C. I. von Meijenfeldt, Maarten J. van der Laan, Clark J. A. M. Zeebregts, Kenneth B. Christopher

**Affiliations:** 1 Department of Surgery (Division of Vascular Surgery), University Medical Center Groningen, University of Groningen, Groningen, The Netherlands; 2 Department of Surgery, Deventer Ziekenhuis, Deventer, The Netherlands; 3 Renal Division, Brigham and Women’s Hospital, The Nathan E. Hellman Memorial Laboratory, Renal Division, Brigham and Women’s Hospital, Boston, Massachusetts, United States of America; National Yang-Ming University, TAIWAN

## Abstract

**Objective:**

Red cell distribution width (RDW) is associated with mortality and bloodstream infection risk in the critically ill. In vascular surgery patients surviving critical care it is not known if RDW can predict subsequent risk of all-cause mortality following hospital discharge. We hypothesized that an increase in RDW at hospital discharge in vascular surgery patients who received critical care would be associated with increased mortality following hospital discharge.

**Design, setting, and participants:**

We performed a two-center observational cohort study of critically ill non-cardiac vascular surgery patients surviving admission 18 years or older treated between November, 1997, and December 2012 in Boston, Massachusetts.

**Exposures:**

RDW measured within 24 hours of hospital discharge and categorized a priori as ≤13.3%, 13.3–14.0%, 14.0–14.7%, 14.7–15.8%, >15.8%.

**Main outcomes and measures:**

The primary outcome was all cause mortality in the 90 days following hospital discharge.

**Results:**

The cohort included 4,715 patients (male 58%; white 83%; mean age 62.9 years). 90 and 365-day post discharge mortality was 7.5% and 14.4% respectively. In the cohort, 47.3% were discharged to a care facility and 14.8% of patients were readmitted within 30 days. After adjustment for age, gender, race, Deyo-Charlson comorbidity Index, patient type, acute organ failures, prior vascular surgery and vascular surgery category, patients with a discharge RDW 14.7–15.8% or >15.8% have an adjusted OR of 90-day post discharge mortality of 2.52 (95%CI, 1.29–4.90; P = 0.007) or 5.13 (95%CI, 2.70–9.75; P <0.001) relative to patients with a discharge RDW ≤13.3%. The adjusted odds of 30-day readmission in the RDW >15.8% group was 1.52 (95%CI, 1.12–2.07; P = 0.007) relative to patients with a discharge RDW ≤13.3%. Similar adjusted discharge RDW-outcome associations are present at 365 days following hospital discharge and for discharge to a care facility.

**Conclusions:**

In critically ill vascular surgery patients who survive hospitalization, an elevated RDW at hospital discharge is a strong predictor of subsequent mortality, hospital readmission and placement in a care facility. Patients with elevated RDW are at high risk for adverse out of hospital outcomes and may benefit from closer post discharge follow-up and higher intensity rehabilitation.

## Introduction

Survivors of critical illness suffer from significant long-term morbidity and mortality [1]. Post-hospital discharge mortality in intensive care unit (ICU) survivors is over 15% at one year and near 40% at three years [2, 3]. Little is known about the post-hospital survival for critically ill vascular surgery patients in general or the specific risk factors for adverse outcomes in this population. The existing studies on risk factors and predictive models of outcomes following vascular surgery are not focused on those requiring critical care [4–7].

Inflammation is central for the initiation and propagation of vascular disease [8]. Vascular surgery patients have high inflammatory activation as surgery induces additional pro-inflammatory cytokines [9]. Inflammatory mediators correlate with Red cell distribution width (RDW), a measure of the variation in circulating red cell size commonly reported in the complete blood count [10–12]. RDW is robustly associated with markers of chronic subclinical inflammation, elevated oxidative stress, malnutrition, C-reactive protein, interleukin-6, erythrocyte sedimentation rate and beta-natriuretic peptide [10–13]. Patients with elevated RDW have a significantly higher risk of peripheral artery disease [14, 15], hypertension [16], and coronary artery disease event rates [17].

Our prior cohort studies in medical and surgical patients demonstrate that RDW is also associated with critical illness outcomes when measured at hospital admission and at hospital discharge [18, 19]. Our critical illness outcome studies did not focus on the vascular surgery population where RDW is associated with vascular disease presence [14–17]. As the vascular surgery population has increased complications, mortality and readmissions following hospital discharge [20], we sought to determine the relationship between RDW at hospital discharge and post-hospital outcomes in vascular surgery patients who required critical care. We hypothesized that elevated RDW at hospital discharge would be associated with increased all cause 90-day post-hospital discharge mortality. To test this hypothesis, we performed a two-center observational cohort study of 4,715 adults who underwent vascular surgery and were treated with critical care and survived hospitalization.

## Materials and methods

### Source population

We extracted administrative and laboratory data from patients admitted to two Boston hospitals: Brigham and Women’s Hospital (BWH), with 777 beds and Massachusetts General Hospital (MGH) with 999 beds. The two hospitals provide primary as well as tertiary care to an ethnically and socioeconomically diverse population within eastern Massachusetts and the surrounding region.

### Data sources

Data on all patients admitted to BWH or MGH between November 2, 1997 and December 31, 2012 were obtained through the Research Patient Data Registry (RPDR), a computerized registry which serves as a central data warehouse for all inpatient and outpatient records at Partners HealthCare sites which includes BWH and MGH. The RPDR has been used for other clinical research studies [18, 19, 21, 22]. This study was approved by the Partners Human Research Committee, the Institutional Review Board (IRB) of Partners HealthCare. Informed consent of study subjects was not obtained. The IRB approval included a waiver of the requirement to obtain informed consent because the risk to study subjects, including risk to privacy, was deemed to be minimal, obtaining informed consent of study subjects was not feasible and the rights and welfare of the subjects would not be adversely affected by the waiver.

### Study population

During the study period there were 7,608 unique patients, age ≥ 18 years, who received critical care, were assigned Current Procedural Terminology (CPT) codes for vascular surgery in the six days prior to ICU admission to 2 days after (S1 Appendix), and were assigned a Diagnostic Related Group code. ICU admission was determined by assignment of the CPT code 99291 (critical care, first 30–74 minutes) during hospitalization admission, a validated approach for ICU admission in the RPDR database [23]. Exclusions included: 3 patients who had white blood cells over 150,000/μl as a high white blood cell count may skew the automatically calculated RDW [24]; 961 patients who died as in-patients; 76 patients with End Stage Renal Disease; and 1,853 patients who did not have RDW drawn within 24 hours of hospital discharge. Thus, 4,715 patients constituted the total study population.

### Exposure of interest and comorbidities

The exposure of interest was RDW within 24 hours of hospital discharge and categorized a priori as ≤13.3%, 13.3–14.0%, 14.0–14.7%, 14.7–15.8%, and >15.8%. For the duration of the study, the RDW was determined directly from the red blood cell histogram and expressed as coefficient of variation (CV) via automated hematology analyzers. Sepsis was defined as the presence of International Classification of Diseases, Ninth Revision, Clinical Modification (ICD-9-CM) codes 038, 995.91, 995.92, or 785.52, 3 days prior to critical care initiation to 7 days after critical care initiation [25]. We utilized the Deyo-Charlson index to assess the burden of chronic illness [26] employing ICD-9-CM coding algorithms which are well studied and validated [27, 28]. History of hypertension was identified using ICD-9-CM codes (401.0, 401.1, 401.9, 405, 405.01, 405.09, 405.1, 405.11, 405.19, 405.9, 405.91, and 405.99) [29]. Patient Type is defined as Medical or Surgical and incorporates the Diagnostic Related Grouping (DRG) methodology [30] and is published by the Centers for Medicare & Medicaid Services (CMS) [31]. Number of organs with failure was adapted from Martin et al [32] and defined by a combination of ICD-9-CM and Current Procedural Terminology (CPT) codes relating to acute organ dysfunction assigned from 3 days prior to critical care initiation to 30 days after critical care initiation [33, 34]. Noncardiogenic acute respiratory failure was identified by the presence of ICD-9-CM codes for respiratory failure or pulmonary edema (518.4, 518.5, 518.81, and 518.82) and mechanical ventilation (96.7×), excluding congestive heart failure (428.0–428.9) following hospital admission [24]. Changes from the expected hospital length of stay (LOS) were computed as the difference between the actual LOS and the geometric mean LOS for each DRG as determined by the Centers for Medicare & Medicaid Services [13].

Patients were considered to have exposure to inotropes and vasopressors if pharmacy records in the 3 days prior to the 7 days after critical care initiation showed evidence of the use of dopamine, dobutamine, epinephrine, norepinephrine, phenylephrine, milrinone or vasopressin. As adding exogenous red blood cells through repeated transfusions is reported to skew the RDW [24], transfusion data was obtained via blood bank reports. The number of packed red blood cell units transfused in the 48 hours prior to critical care initiation through the hospital stay was recorded.

### Assessment of mortality

Information on vital status for the study cohort was obtained from the Social Security Administration Death Master File. The accuracy of the Social Security Administration Death Master File for in-hospital and out of hospital mortality in our administrative database is validated [23]. 100% of the cohort had vital status present at 365 days following hospital discharge.

### Study outcomes

The primary outcome was all-cause 90-day post-discharge mortality. Secondary outcomes included 365-day post-discharge mortality, unplanned 90-day hospital readmission [35, 36] and discharge to a care facility. Hospital readmission was determined from RPDR hospital admission data as previously described [37] and defined as a subsequent or unscheduled admission to BWH or MGH following the index hospitalization associated with the critical care exposure [37–39]. We excluded readmissions with DRG codes that are commonly associated with planned readmissions in addition to DRGs for transplantation, procedures related to pregnancy, and psychiatric issues [3, 37]. Discharge care facility data was determined from hospital records [40].

### Power calculations and statistical analysis

Based on our prior work [19], we assume that absolute 90-day post-hospital discharge mortality will increase 12.7% in patients with discharge RDW >15.8% compared to those with discharge RDW ≤13.3%. Using Stata 14.1MP statistical software (College Station, TX), we estimated the sample size for two-sample comparison of proportions. With an alpha error level of 5% (two-sided) and a power of 90%, the sample size thus required for our primary end point (90-day post-hospital discharge mortality) is 114 in the RDW >15.8% group and 114 in the RDW ≤13.3% group.

Categorical variables were described by frequency distribution, and compared across RDW groups using contingency tables and chi-square testing. Continuous variables were examined graphically (histogram, box plot) and in terms of summary statistics (mean, standard deviation, median, interquartile range), and then compared across exposure groups using one-way analysis of variance (ANOVA). Unadjusted associations between covariates and mortality were estimated by bivariable logistic regression models. Adjusted odds ratios were estimated by multivariable logistic regression models with inclusion of a priori determined covariate terms thought to plausibly associate with both RDW levels and 90-day post-discharge mortality. Overall model fit was assessed using the Hosmer Lemeshow test.

We assessed possible effect modification of sepsis, creatinine, white blood count, hematocrit and transfusion on the risk of mortality using the likelihood-ratio test. We evaluated for confounding by individually running the adjusted model with and without terms for creatinine, white blood count, transfusion, hematocrit, vasopressors/inotropes or mechanical ventilation. Receiver operator characteristic (ROC) curves were constructed to analyze the discriminating power of discharge RDW for predicting 90-day post-discharge mortality, and the areas under each ROC curve were compared. An empirical estimation of the optimal ROC cutoff point was performed with the cutpt command in Stata utilizing bootstrapping [41]. The continuous crude and adjusted relationship between discharge RDW level and risk of 90-day post-discharge mortality was graphically represented utilizing the coefplot command [42]. All p-values are two-tailed, with values <0.05 were considered statistically significant. All analyses were performed using Stata 14.1MP statistical software (College Station, TX).

## Results

Table 1 shows characteristics of the study population. Most patients were male (58%), white (83%) and the majority had surgically related DRGs (84%). The mean age at hospital admission was 62.6 (SD 16.9) years. Post-hospital discharge mortality rates were 3.9% at 30-days, 7.5% at 90 days and 14.4% at 365 days. 90-day readmission rate was 23%. The vascular surgery procedure classes in the cohort included abdomen (36%), amputations (2%), compartment syndrome (2%), lower extremity (8%), neck (8%), upper extremity (31%) and venous (13%). Fifty-four percent of the vascular procedures were endovascular. Age, Deyo-Charlson Index, acute organ failure, malignancy, acute kidney injury, sepsis, RDW at hospital discharge, change in expected length of stay, discharge to care facility and hospital readmission are significant predictors of 90-day mortality (Table 1).

**Table 1:** Characteristics and Unadjusted association of potential prognostic determinants with 90-day post discharge mortality^a^.

Characteristics	Alive N = 4,361	Expired^a^ N = 354	Total N = 4,715	P-value	Unadjusted OR (95%CI) for 90-day Post Discharge Mortality
*Age years-mean***±***SD*	61.8 ± 16.9	71.9 ± 13.2	62.6 ± 16.9	<0.001^†^	1.05 (1.04, 1.06)
*Male Gender-no*.*(%)*	2,528 (58)	194 (55)	2,722 (58)	0.25	0.88 (0.71, 1.09)
*Non-White Race-no*.*(%)*	768 (18)	52 (15)	820 (17)	0.16	0.81 (0.59, 1.09)
*Surgical Patient Type-no*.*(%)*	3,666 (84)	296 (84)	3,962 (84)	0.83	0.97 (0.72, 1.30)
*Prior Vascular Surgery-no*.*(%)*	529 (12)	50 (14)	579 (12)	0.27	1.19 (0.87, 1.63)
*Deyo-Charlson index-no*.*(%)*				<0.001	
0–1	1,012 (23)	25 (7)	1,037 (22)		1.00 (Referent)
2–3	1,914 (44)	104 (29)	2,018 (43)		2.20 (1.41, 3.43)
4–6	1,230 (28)	179 (51)	1,409 (30)		5.89 (3.84, 9.03)
≥7	205 (5)	46 (13)	251 (5)		9.08 (5.46, 15.12)
*Number of organs with acute failure-no*.*(%)*				<0.001	
0	1,309 (30)	48 (14)	1,357 (29)		1.00 (Referent)
1	1,546 (35)	124 (35)	1,670 (35)		2.19 (1.55, 3.08)
2	950 (22)	107 (30)	1,057 (22)		3.07 (2.16, 4.36)
3	385 (9)	52 (15)	437 (9)		3.68 (2.45, 5.54)
≥4	171 (4)	23 (7)	194 (4)		3.67 (2.18, 6.18)
*Malignancy-no*.*(%)*	731 (17)	136 (38)	867 (18)	<0.001	3.10 (2.47, 3.89)
*Hypertension-no*.*(%)*	976 (22)	39 (11)	1,015 (22)	<0.001	0.43 (0.31, 0.60)
*Acute Kidney Injury-no*.*(%)* ^b^	247 (7)	38 (14)	285 (7)	<0.001	2.38 (1.65, 3.43)
*Sepsis-no*.*(%)*	188 (4)	31 (9)	219 (5)	<0.001	2.13 (1.43, 3.17)
*Noncardiogenic acute respiratory failure-no*.*(%)*	387 (9)	30 (8)	417 (9)	0.80	0.95 (0.64, 1.40)
*Vasopressors/Inotropes-no*.*(%)*	2,203 (51)	167 (47)	2,370 (50)	0.23	0.88 (0.70, 1.09)
*Acute Organ Failure Score-mean***±***SD* ^c^	8.0 ± 3.8	10.8 ± 3.6	8.2 ± 3.8	<0.001^†^	1.20 (1.17, 1.24)
*RDW at Hospital Discharge-mean***±***SD*	14.8 ± 1.7	16.2 ± 2.1	14.9 ± 1.8	<0.001^†^	1.38 (1.31, 1.44)
*Change in Expected Length of Stay-median*[IQR]	3.7 [0.4, 10.4]	8.5 [1.1, 19.4]	4.0 [0.4, 11]	<0.001^‡^	1.02 (1.02, 1.03)
*Discharge to Care Facility-no*.*(%)*	1,991 (89)	235 (11)	2,226 (47)	<0.001	2.35 (1.87, 2.95)
90-Day Readmission-no.(%)	965 (22)	121 (34)	1,086 (23)	<0.001	1.83 (1.45, 2.30)

Data presented as no. (%) unless otherwise indicated. P determined by chi-square except for † determined by ANOVA or ‡ determined by Kruskal-Wallis test.

^a.^ Expired within 90-days following hospital discharge

^b.^ Acute Kidney Injury is RIFLE class injury or failure and available on 3,989 patients.

^c.^ The Acute Organ Failure score is a severity of illness risk-prediction score ranging from 0–30 points with 30 having the highest risk for mortality

Patient characteristics of the study cohort were stratified according to discharge RDW levels (Table 2).

**Table 2:** Patient characteristics by RDW category group.

Characteristic	Discharge RDW					
	≤13.3	13.3–14.0	14.0–14.7	14.7–15.8	>15.8	P-value
N	692	1,023	885	949	1,166	
*Age-mean****±****SD*	55.72 ± 17.83	61.92 ± 16.39	64.43 ± 16.56	63.91 ± 17.23	62.57 ± 16.87	<0.001^†^
*Male Gender-no*.*(%)*	454 (66)	624 (61)	502 (57)	544 (57)	2,722 (58)	<0.001
*Non-White Race-no*.*(%)*	122 (18)	164 (16)	148 (17)	170 (18)	820 (17)	0.59
*Surgical Patient Type-no*.*(%)*	488 (71)	828 (81)	780 (88)	848 (89)	3,962 (84)	<0.001
*Prior Vascular Surgery-no*.*(%)*	52 (8)	98 (10)	110 (12)	128 (13)	191 (16)	<0.001
*Endovascular-no*.*(%)*	442 (64)	599 (59)	458 (52)	436 (46)	614 (53)	<0.001
*Deyo-Charlson index-no*.*(%)*						<0.001
0–1	269 (39)	260 (25)	171 (19)	186 (20)	151 (13)	
2–3	328 (47)	477 (47)	408 (46)	400 (42)	405 (35)	
4–6	87 (13)	255 (25)	264 (30)	309 (33)	494 (42)	
≥7	8 (1)	31 (3)	42 (5)	54 (6)	116 (10)	
*Number of organs with acute failure -no*.*(%)*						<0.001
0	348 (50)	390 (38)	243 (27)	182 (19)	194 (17)	
1	248 (36)	396 (39)	328 (37)	318 (34)	380 (33)	
2	69 (10)	174 (17)	216 (24)	282 (30)	316 (27)	
3	23 (3)	50 (4.89)	70 (8)	116 (12)	178 (15)	
≥4	4 (1)	13 (1)	28 (3)	51 (5)	98 (8)	
*Malignancy-no*.*(%)*	73 (11)	140 (14)	143 (16)	197 (21)	314 (27)	<0.001
*Hypertension-no*.*(%)*	94 (14)	202 (20)	216 (24)	219 (23)	284 (24)	<0.001
*Acute Kidney Injury-no*.(%)*	12 (2)	25 (3)	38 (5)	67 (9)	143 (15)	<0.001
*Sepsis-no*.*(%)*	4 (1)	15 (1)	22 (2)	55 (6)	219 (5)	<0.001
*Noncardiogenic acute respiratory failure -no*.*(%)*	36 (5)	72 (7)	71 (8)	105 (11)	417 (9)	<0.001
*Vasopressors/Inotropes -no*.*(%)*	237 (34)	483 (47)	438 (49)	548 (58)	2,370 (50)	<0.001
*Acute Organ Failure Score-mean***±***SD*	6.61 ± 3.53	7.43 ± 3.58	8.12 ± 3.55	8.75 ± 3.79	8.23 ± 3.83	<0.001
*Change in Expected Length of Stay-median*[IQR]	1.4 [-1.4, 4.4]	2.4 [-0.1, 6.4]	3.9 [0.6, 9.5]	6.4 [1.5, 15]	8.9 [2.2, 21.9]	<0.001^‡^
*Discharge to Care Facility-no*.*(%)*	187 (27)	386 (38)	417 (47)	512 (54)	724 (62)	<0.001
*90-day Readmission-No*.*(%)*	112 (16)	178 (17)	193 (22)	257 (27)	346 (30)	<0.001
*90-day post-discharge Mortality-no*.*(%)*	11 (2)	37 (4)	49 (6)	71 (7)	186 (16)	<0.001
*365-day post-discharge Mortality-no*.*(%)*	35 (5)	77 (8)	103 (12)	142 (15)	320 (27)	<0.001

Data presented as n (%) unless otherwise indicated. P determined by chi-square except for ^†^ determined by ANOVA or ^‡^ determined by Kruskal-Wallis test.

* Acute Kidney Injury is RIFLE class injury or failure. Information on acute kidney injury available on 3,898 patients

Most factors significantly differed between stratified groups including 90-day post-discharge mortality.

### Primary outcome

**Fig 1:**
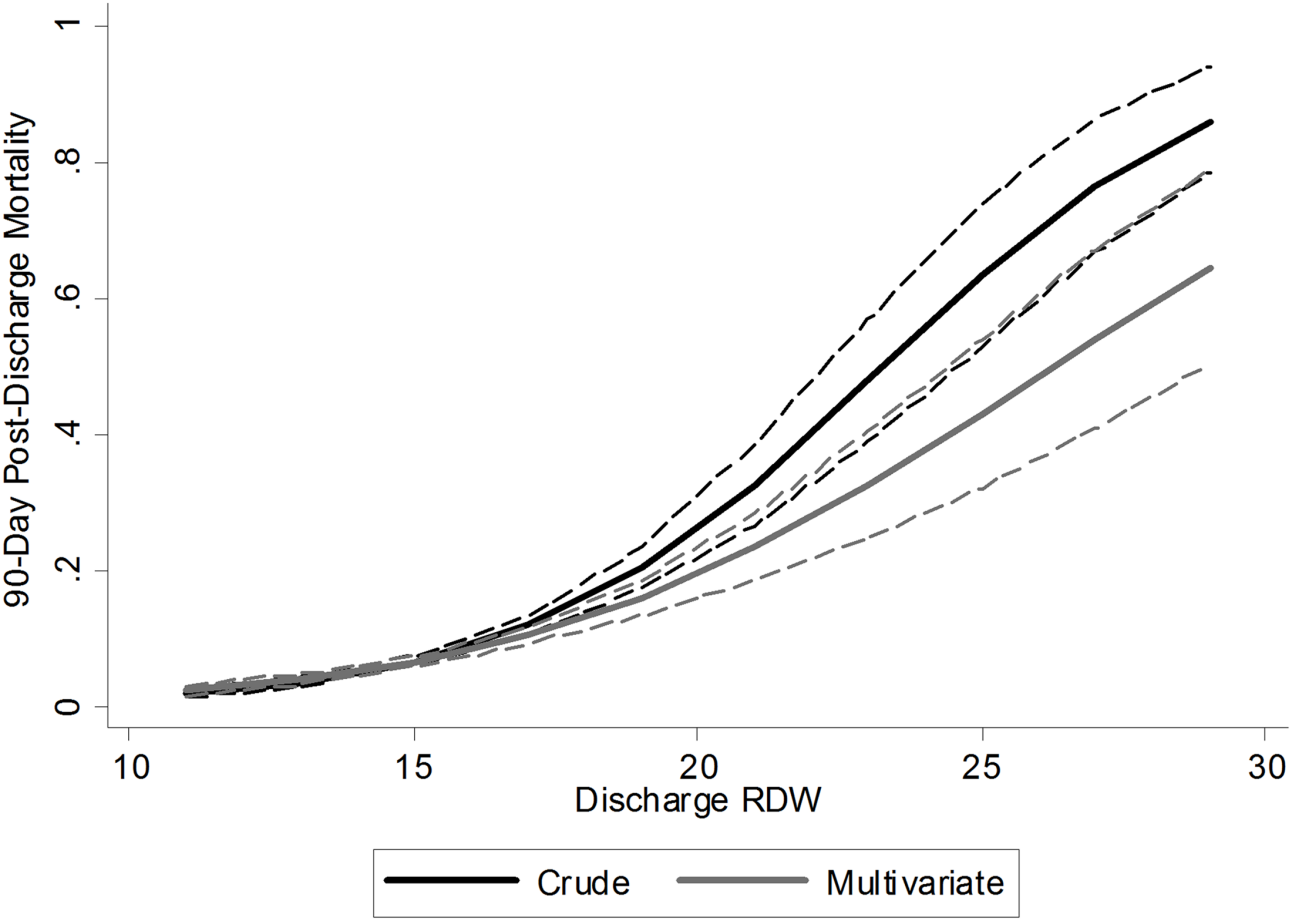
Coefficient plot. Plot representing crude (black) and multivariate (grey) estimates of the discharge RDW-mortality association with confidence intervals (dashes). Multivariate estimates adjusted for age, race, patient type, Deyo-Charlson index, Number of organs with acute failure, sepsis, prior vascular surgery and vascular surgery class.

RDW at hospital discharge was a strong predictor of 90-day post-discharge mortality following multivariable adjustment for relevant confounders (Fig 1 and Table 3).

**Table 3:** Unadjusted and adjusted associations between RDW category and 90-day post-discharge mortality (N = 4,715).

	≤13.3	13.3–14.0	14.0–14.7	14.7–15.8	>15.8	AUC	HL-χ^2^
90-day post-discharge mortality	OR (95% CI) P	OR (95% CI) P	OR (95% CI) P	OR (95% CI) P	OR (95% CI) P		
Crude	1.00 (Referent)^a^	2.32 (1.18, 4.59) 0.015	3.63 (1.87, 7.03) <0.001	5.01 (2.63, 9.52) <0.001	11.75 (6.35, 21.76) <0.001	0.70	0.99
Adjusted^b^	1.00 (Referent)^a^	1.61 (0.81, 3.21) 0.18	2.09 (1.06, 4.12) 0.033	2.52 (1.29, 4.90) 0.007	5.13 (2.70, 9.75) <0.001	0.80	0.33
Adjusted^c^	1.00 (Referent)^a^	1.65 (0.83, 3.31) 0.16	2.21 (1.12, 4.37) 0.022	2.62 (1.35, 5.12) 0.005	5.27 (2.77, 10.05) <0.001	0.82	0.77
Adjusted^d^	1.00 (Referent)^a^	1.62 (0.81, 3.26) 0.17	1.99 (1.00, 3.96) 0.049	2.36 (1.21, 4.62) 0.012	4.49 (2.34, 8.60) <0.001	0.82	0.13

Note: AUC is the area under the receiver operating characteristic curve; HL- χ^2^ is the Hosmer-Lemeshow χ^2^ goodness-of-fit test; BIC is Bayesian information criterion

^a.^ Referent in each case is RDW≤13.3

^b.^ Model 1: Estimates adjusted for age, race, patient type, Deyo-Charlson index, Number of organs with acute failure, sepsis, prior vascular surgery and vascular surgery class.

^c.^ Model 2: Estimates adjusted for age, race, patient type, Deyo-Charlson index, hypertension, Number of organs with acute failure, sepsis, prior vascular surgery and vascular surgery class.

^d.^ Model 3: Estimates adjusted for covariates in Model 1 and additionally for change in expected length of stay, malignancy and calendar quarter.

In model 1 adjusting for age, race, patient type, Deyo-Charlson index, Number of organs with acute failure, sepsis, prior vascular surgery and vascular surgery class, the odds of 90-day mortality in the RDW 14.7–15.8% and RDW >15.8% groups was 5 and 12-fold higher than the RDW ≤ 13.3% group respectively. Discharge RDW remained a significant predictor of odds of mortality after adjustment for age, gender, race, DRG type, Deyo-Charlson index, acute organ failure, sepsis, prior vascular surgery and vascular surgery category. The adjusted odds of 90-day mortality in the RDW 14.7–15.8% and RDW >15.8% groups was 2.5 and 5-fold respectively that of those with RDW ≤ 13.3%. The AUC for the prediction model 1 for 90 day post-discharge mortality was 0.80 (95%CI 0.78–0.83). The prediction model 1 showed good calibration (HL χ^2^ 9.2, P = 0.33) (Table 3). The optimal cut point for 90-day post-discharge mortality was RDW = 15.45 (95%CI 15.01–15.88).

There was no significant effect modification of the RDW-90-day post-discharge mortality association on the basis of sepsis (P-interaction = 0.23), hospital (P-interaction = 0.09), chronic kidney disease (P-interaction = 0.52) or transfusion (P-interaction = 0.48). Though the effect sizes differed, the direction of the estimates and overall significance of the RDW-post-discharge mortality association was not materially altered by hospital. Additional adjustment of the model for hospital, endovascular repair, calendar quarter of hospital discharge or Red Blood Cell Transfusions did not materially alter the point estimates. Similar robust crude and adjusted discharge RDW-mortality associations are present at 365 days following hospital discharge (data not shown).

### Secondary outcomes

The adjusted odds of 90-day readmission in the RDW 14.7–15.8% and RDW >15.8% groups was 1.9 and 2.1 fold higher respectively that of those with RDW ≤ 13.3% (Table 4).

**Table 4:** Unadjusted and adjusted associations between RDW category, discharge to facility and hospital readmission (N = 4,715).

	≤13.3	13.3–14.0	14.0–14.7	14.7–15.8	>15.8
	OR (95% CI) P	OR (95% CI) P	OR (95% CI) P	OR (95% CI) P	OR (95% CI) P
30-day hospital readmission					
Crude	1.00 (Referent)^a^	0.97 (0.71, 1.32) 0.83	1.13 (0.83, 1.54) 0.44	1.60 (1.19, 2.13) 0.002	2.02 (1.53, 2.66) <0.001
Adjusted^b^	1.00 (Referent)^a^	0.91 (0.66, 1.24) 0.55	0.99 (0.72, 1.37) 0.96	1.32 (0.96, 1.80) 0.084	1.53 (1.12, 2.07) 0.007
90-day hospital readmission					
Crude	1.00 (Referent)^a^	1.09 (0.84, 1.41) 0.51	1.44 (1.12, 1.87) 0.005	1.92 (1.50, 2.46) <0.001	2.19 (1.72, 2.77) <0.001
Adjusted^b^	1.00 (Referent)^a^	1.04 (0.80, 1.35) 0.78	1.29 (0.98, 1.69) 0.066	1.61 (1.24, 2.10) <0.001	1.66 (1.27, 2.16) <0.001
Discharge to Facility					
Crude	1.00 (Referent)^a^	1.64 (1.33, 2.02) <0.001	2.41 (1.94, 2.98) <0.001	3.16 (2.56, 3.91) <0.001	4.42 (3.60, 5.43) <0.001
Adjusted^b^	1.00 (Referent)^a^	1.26 (1.01, 1.58) 0.043	1.50 (1.19, 1.89) 0.001	1.70 (1.35, 2.15) <0.001	2.10 (1.67, 2.65) <0.001

^a.^ Referent in each case is RDW≤13.3

^b.^ Estimates adjusted for age, race, patient type, Deyo-Charlson index, Number of organs with acute failure, sepsis, prior vascular surgery and vascular surgery class.

Further, the adjusted odds of discharge to a facility rather than to home (i.e., rehabilitation and long-term acute care) in the RDW 14.7–15.8% and RDW > 15.8% groups was 1.7 and 2.1 fold higher, respectively, compared with RDW ≤ 13.3% group (Table 4).

## Discussion

The main finding of our study is the graded relationship between increased RDW at hospital discharge in vascular surgery patients who survive critical care and adverse outcomes following hospital discharge. Elevated RDW at hospital discharge is significantly associated with 90-day mortality following adjustment for potential confounders. Further, discharge RDW is associated with placement in a care facility and 90-day unplanned hospital readmission. Elevated RDW at hospital discharge may identify patients who are at a high risk for adverse outcomes following hospital discharge.

Existing studies on the risk factors for non-cardiac vascular surgery outcomes focus on short-term and in-hospital mortality and emphasize demographics, clinical variables and the American Society of Anesthesiologist score [4, 43, 44]. Studies on post-hospital outcomes in vascular surgery are emerging and include the identification of risk factors and consequences of hospital readmission [45–48], surgical site infections [49] and unplanned reoperations [50]. In surgical ICU survivors, elevated RDW at hospital discharge is significantly associated with out of hospital mortality [19].

Biomarkers of systemic inflammation are commonly elevated in vascular surgery patients and are also correlated with elevated RDW [10–13]. Inflammation alters erythropoiesis via increased red cell apoptosis, erythroid precursor myelosuppression, decreased erythropoietin production, decreased iron bioavailability, and erythropoietin resistance [51–54]. In critical illness survivors erythropoiesis suppression persists in combination with hypo-active bone-marrow from ongoing inflammation [38]. Ultimately inflammation and oxidative stress alter erythrocyte homeostasis resulting in decreased RBC survival time, greater variations in RBC cell sizes and a higher RDW.

RDW is a widely available and cost-effective laboratory test commonly measured within a complete blood count. Patients with an elevated RDW at hospital discharge may benefit from more intensive follow-up regimes in the outpatient clinic and at rehabilitation. Also, discharge RDW may be valuable as prognostic information in patient-provider discussions regarding goals and palliative services. The low cost, wide availability, quick turn-around time, and facile interpretation of the RDW would tend to favor its adoption over more cumbersome and subjective assessments in these circumstances.

Our study may be limited by potential unmeasured confounding, residual confounding and reverse causation. The generalizability of our results may be limited as our cohort is from two academic medical centers from the same region. The limitations of RDW include the formula [RDW-CV = 1 SD ÷ (MCV × 100)], the time since blood draw and RDW determination and RDW elevations in the context of iron, folate or B12 deficiency related anemia, as well as Sickle cell disease, and Myelodysplastic syndrome [55]. Further, we can only include readmissions to the two hospitals under study. Though these hospitals have a large regional catchment we cannot but account for all readmissions to all hospitals. Reliance on ICD-9-CM codes to determine covariates may underestimate the true incidence or prevalence [56]. Excluding particular variables from our a priori regression model that are associated with the outcome may introduce bias by not adjusting for confounding of that particular variable. The strengths of the study include large sample size, adequate study power and complete follow-up for mortality at 365 days.

## Conclusion

In vascular surgery patients surviving critical illness, the red cell distribution width at hospital discharge is a predictor of out of hospital mortality and hospital readmission. Vascular surgery patients with elevated RDW at discharge are at high-risk for subsequent adverse outcomes. Measuring RDW at hospital discharge is inexpensive and may provide a cost-effective way to identify patients at high risk for subsequent adverse outcomes. This study provides support for future vascular surgery investigations to consider adding RDW to other established predictive models to stratify critically ill vascular surgery patients at risk for adverse events.

## Supporting information

S1 Appendix Supplemental methods. Current Procedural Terminology (CPT) codes utilized to define vascular surgery.
(DOCX)
