# Supplementary material for: Red cell distribution width at hospital discharge and out-of hospital outcomes in critically ill non-cardiac vascular surgery patients
Source: PLoS One. 2018 Sep 5;13(9):e0199654. doi: 10.1371/journal.pone.0199654 (PMC6124728; doi:10.1371/journal.pone.0199654)
Supplement: S1 Appendix — Current Procedural Terminology (CPT) codes utilized to define vascular surgery. (DOCX) [file pone.0199654.s001.docx]

**S1 Appendix**

**Supplemental Methods**

**Neck**

*Endovascular*

34001, 36100, 37195

*Open*

35001, 35002, 35005, 35180, 35188, 35201, 35231, 35261, 35301, 35390, 35501, 35506, 35508, 35509, 35601, 35606, 35642, 35691, 35693, 35695, 35694, 35701, 35800, 35901, 37600, 37605, 37606, 37609, 37615.

**Upper extremity**

*Endovascular*

0033T, 0034T, 34101, 34111, 35458, 35475, 35484, 35494, 36120, 36140, 36145, 36215, 36216, 36217, 36218, 36870

*Open*

35011, 35013, 35045, 35206, 35207, 35236, 35266, 35311, 35321, 35507, 35511, 35515, 35516, 35518, 35521, 35526, 35612, 35616, 35621, 35623, 35626, 35645, 35650, 35875, 35876, 36819, 36820, 36821, 36825, 36830, 36831, 36832, 36833, 36834, 37607

**Thorax**

*Endovascular*

0035T, 0036T, 0037T, 34051, 36013

**Compartment syndrome**

*Upper extremity*

24495, 25020, 25023

*Lower extremity*

27025, 27600, 27601, 27602, 27892, 27893, 27894

**Amputations**

*Upper extremity*

23900, 23920, 23921, 24900, 24920, 24925, 24930, 24931, 25900, 25905, 25907, 25909, 25915, 25920, 25922, 25924, 25927, 25929, 25931, 26910, 26951, 26952

*Lower extremity*

27290, 27295, 27590, 27591, 27592, 27594, 27596, 27598, 27880, 27881, 27882, 27884, 27886, 27888, 27889, 28800, 28805, 28810, 28820, 28825

**Abdomen**

*Endovascular*

34151, 34800, 34802, 34803, 34804, 34805, 34808, 34813, 34820, 34825, 34826, 34900, 35400, 35450, 35452, 35454, 35471, 35472, 35473, 35480, 35481, 35482, 35490, 35492, 36160, 36200, 36245, 36246, 36247, 36248

*Open*

34830, 34831, 34832, 34833, 34834, 35081, 35082, 35091, 35092, 35102, 35103, 35111, 35112, 35121, 35122, 35131, 35132, 35182, 35189, 35221, 35251, 35281, 35331, 35341, 35351, 35355, 35361, 35363, 35531, 35536, 35541, 35546, 35548, 35549, 35551, 35560, 35563, 35565, 35631, 35636, 35651, 35641, 35646, 35647, 35663, 35665, 35840, 35870, 35907, 37617, 37799,

**Lower extremity**

*Endovascular*

34201, 34203, 35456, 35459, 35470, 35474, 35483, 35485, 35493, 35495, 36002, 37201, 37202, 37203, 37204, 37205, 37206, 37207, 37208, 37209, 37250, 37251

*Open*

34812, 35141, 35142, 35151, 35152, 35184, 35190, 35226, 35256, 35286, 35371, 35372, 35381, 35533, 35556, 35558, 35566, 35571, 35583, 35585, 35587, 35654, 35656, 35661, 35666, 35671, 35681, 35682, 35683, 35685, 35686, 35700, 35721, 35741, 35761, 35860, 35879, 35881, 35903, 37618

**Venous**

34401, 34421, 34451, 34471, 34490, 34501, 34502, 34510, 34520, 34530, 35460, 35476, 36005, 36011, 36012, 36468, 36469, 36470, 36471, 37140, 37145, 37160, 37180, 37181, 37620, 37650, 37660, 37720, 37730, 37735, 37760, 37780, 37785, 37500, 37565, 36010, 36800, 36810, 36815, 36835, 36860, 36861, 36014, 36015, 36822, 36823
